# Osmolality and Non-Structural Carbohydrate Composition in the Secondary Phloem of Trees across a Latitudinal Gradient in Europe

**DOI:** 10.3389/fpls.2016.00726

**Published:** 2016-06-01

**Authors:** Anna Lintunen, Teemu Paljakka, Tuula Jyske, Mikko Peltoniemi, Frank Sterck, Georg von Arx, Hervé Cochard, Paul Copini, Maria C. Caldeira, Sylvain Delzon, Roman Gebauer, Leila Grönlund, Natasa Kiorapostolou, Silvia Lechthaler, Raquel Lobo-do-Vale, Richard L. Peters, Giai Petit, Angela L. Prendin, Yann Salmon, Kathy Steppe, Josef Urban, Sílvia Roig Juan, Elisabeth M. R. Robert, Teemu Hölttä

**Affiliations:** ^1^Department of Forest Sciences, University of HelsinkiHelsinki, Finland; ^2^Natural Resources Institute FinlandVantaa, Finland; ^3^Forest Ecology and Forest Management Group, Department of Environmental Sciences, Wageningen UniversityWageningen, Netherlands; ^4^Swiss Federal Institute for Forest, Snow and Landscape Research WSLBirmensdorf, Switzerland; ^5^INRA, UMR 547 PIAF, Université Clermont AuvergneClermont-Ferrand, France; ^6^Alterra, Wageningen University and Research CentreWageningen, Netherlands; ^7^Forest Research Centre, School of Agriculture, University of LisbonLisbon, Portugal; ^8^INRA, University of Bordeaux, UMR BIOGECOTalence, France; ^9^Department of Forest, Botany, Dendrology and Geobiocenology, Mendel University in BrnoBrno, Czech Republic; ^10^Department Territorio e Sistemi Agro-Forestali, Legnaro (PD), Università degli Studi di PadovaPadova, Italy; ^11^Department of Physics, University of HelsinkiHelsinki, Finland; ^12^Laboratory of Plant Ecology, Department of Applied Ecology and Environmental Biology, Ghent UniversityGent, Belgium; ^13^Centre for Ecological Research and Forestry Applications (CREAF)Cerdanyola del Vallès, Spain; ^14^Laboratory of Plant Biology and Nature Management (APNA), Vrije Universiteit BrusselBrussels, Belgium; ^15^Laboratory of Wood Biology and Xylarium, Royal Museum for Central Africa (RMCA)Tervuren, Belgium

**Keywords:** hexose, osmotic concentration, phloem water content, pinitol, raffinose, sucrose, starch

## Abstract

Phloem osmolality and its components are involved in basic cell metabolism, cell growth, and in various physiological processes including the ability of living cells to withstand drought and frost. Osmolality and sugar composition responses to environmental stresses have been extensively studied for leaves, but less for the secondary phloem of plant stems and branches. Leaf osmotic concentration and the share of pinitol and raffinose among soluble sugars increase with increasing drought or cold stress, and osmotic concentration is adjusted with osmoregulation. We hypothesize that similar responses occur in the secondary phloem of branches. We collected living bark samples from branches of adult *Pinus sylvestris, Picea abies, Betula pendula* and *Populus tremula* trees across Europe, from boreal Northern Finland to Mediterranean Portugal. In all studied species, the observed variation in phloem osmolality was mainly driven by variation in phloem water content, while tissue solute content was rather constant across regions. Osmoregulation, in which osmolality is controlled by variable tissue solute content, was stronger for *Betula* and *Populus* in comparison to the evergreen conifers. Osmolality was lowest in mid-latitude region, and from there increased by 37% toward northern Europe and 38% toward southern Europe due to low phloem water content in these regions. The ratio of raffinose to all soluble sugars was negligible at mid-latitudes and increased toward north and south, reflecting its role in cold and drought tolerance. For pinitol, another sugar known for contributing to stress tolerance, no such latitudinal pattern was observed. The proportion of sucrose was remarkably low and that of hexoses (i.e., glucose and fructose) high at mid-latitudes. The ratio of starch to all non-structural carbohydrates increased toward the northern latitudes in agreement with the build-up of osmotically inactive C reservoir that can be converted into soluble sugars during winter acclimation in these cold regions. Present results for the secondary phloem of trees suggest that adjustment with tissue water content plays an important role in osmolality dynamics. Furthermore, trees acclimated to dry and cold climate showed high phloem osmolality and raffinose proportion.

## Introduction

Plants have to keep osmolality levels sufficiently high in the phloem to maintain basic cell metabolism processes (see Rodríguez-Calcerrada et al., 2015) and cell turgor at levels that allow growth (Kröger et al., 2011). Phloem osmolality levels also have a role in various physiological processes in plants e.g., biomass accumulation (Simard et al., 2013; Steppe et al., 2015), control of transpiration (Schroeder et al., 2001), and maintaining xylem hydraulic integrity (Sala et al., 2012; Sevanto et al., 2014; Pfautsch et al., 2015). In addition, high osmolality decreases the wilting point (Bartlett et al., 2012a,b; Charrier et al., 2013a,b) and the ice nucleation temperature (Burke et al., 1976) of living cells thus affecting their ability to tolerate drought and freezing temperatures.

Plants may vary in phloem osmolality because they differ in the control of sugar concentrations (or other osmotic substances), or they differ in phloem water content, i.e., cell osmolality can be increased either by an increase in the amount of solutes or a decrease in the amount of water in the cell. In dry climates, the maintenance of cell turgor may require higher osmolality to compensate for low stem water potentials. In cold climate, such as in the boreal zone, high osmolality and high carbon storage may both be required to avoid symplastic freezing during winters. So far, such processes have been studied for leaves (e.g., O'Neill, 1983; Gross and Koch, 1991; Callister et al., 2008; Bartlett et al., 2014; O'Brien et al., 2014; Maréchaux et al., 2015) but only scarcely for the secondary phloem in plant stems or branches. It has been shown that sucrose concentration in the secondary phloem of *Picea abies* increases with increasing latitude in Finland (Jyske et al., 2015). Moreover, the comparison of studies suggests that the sugar concentration in the secondary phloem increases with increasing elevation in *Larix decidua* (Hoch et al., 2003; Streit et al., 2013), but we lack empirical tests over continental scale on the ability of secondary phloem of trees to osmotically adjust to different climates.

Studies for the osmolality and non-structural carbohydrate (NSC) concentration in the secondary phloem are needed, because the secondary phloem is structurally different from the primary phloem in leaves. The secondary phloem includes non-collapsed and collapsed tissue. Non-collapsed tissue is typically the youngest part of the phloem, whereas older layers in the outer part of the secondary phloem collapse and become storage tissue (Evert, 2006). Sugars are transported between loading (at C sources) and unloading sites (at C sinks) in sieve elements in the non-collapsed phloem tissue. The transport is driven by a gradient in osmotically established turgor pressure (Münch, 1930; Thompson, 2006; De Schepper et al., 2013), and is coordinated with the axial gradient of water potential developed along the xylem compartments (Hölttä et al., 2006). Secondary phloem also needs to tolerate seasonal drought and cold stresses, whereas these stresses can be avoided in the leaves of deciduous species by shedding.

Phloem osmolality is a measure of the moles of solute per kilogram of solvent (mol kg^−1^), and there are different types of solutes that contribute to it: soluble sugars, ions and amino acids. Sucrose is a soluble sugar that is considered as the most important compound being translocated in phloem elements (Pate, 1976). Hexoses (i.e., glucose and fructose) are present in high amounts in all living cells, and can also be important transport sugars in the phloem for some species (Van Bel and Hess, 2008). Raffinose and pinitol occur in small amounts in phloem, but may contribute to protecting cells against environmental stress, such as drought and low temperatures (Bohnert and Shen, 1999; Zuther et al., 2004; Deslauriers et al., 2014). Starch, which is the most common storage form of non-soluble carbohydrates, contributes only marginally to the value of osmolality due to its high molar mass.

Non-structural carbohydrates (i.e., soluble sugars and starch) are constantly transformed from one form to another. Starch, for example, is formed when high levels of soluble sugars occur, and is transformed to sugars if sugar content is low (Escobar-Gutiérrez et al., 1998). Amount and composition of NSC in phloem tissue show a seasonal behavior in temperate and boreal regions (Hoch et al., 2003; Simard et al., 2013; Jyske et al., 2015), and have an important role in the development of cold hardiness: starch is converted into sugars during cold acclimation (Zwieniecki et al., 2015). Furthermore, a fraction of NSCs can be converted to defensive chemicals in some species (Kozlowski, 1992).

In this study, we aimed at showing variation in osmolality and non-structural carbohydrate composition in secondary branch phloem across a large geographical and climatic gradient. We hypothesize that (i) variability in osmolality is mainly controlled by solute content over large geographical scale, (ii) osmolality and solute content increase from the mid-latitudes toward the more drought-prone lower latitudes as well as to more cold-stressed higher latitudes, and locally from moist to dry soil sites, and (iii) the share of raffinose and pinitol among soluble sugars increase from the mid-latitudes toward south and north given their role in tolerating drought and cold stress. To test these hypotheses we collected branches from four widely distributed species *Pinus sylvestris, Picea abies, Betula pendula* and *Populus tremula* from moist and dry soil sites, from boreal, temperate and Mediterranean regions across Europe. For phloem samples of those branches, we analyzed osmolality, concentrations of different sugars and water content, and discuss patterns across the studied geographic/climatic gradient.

## Materials and methods

### Plant material

**Figure 1:**
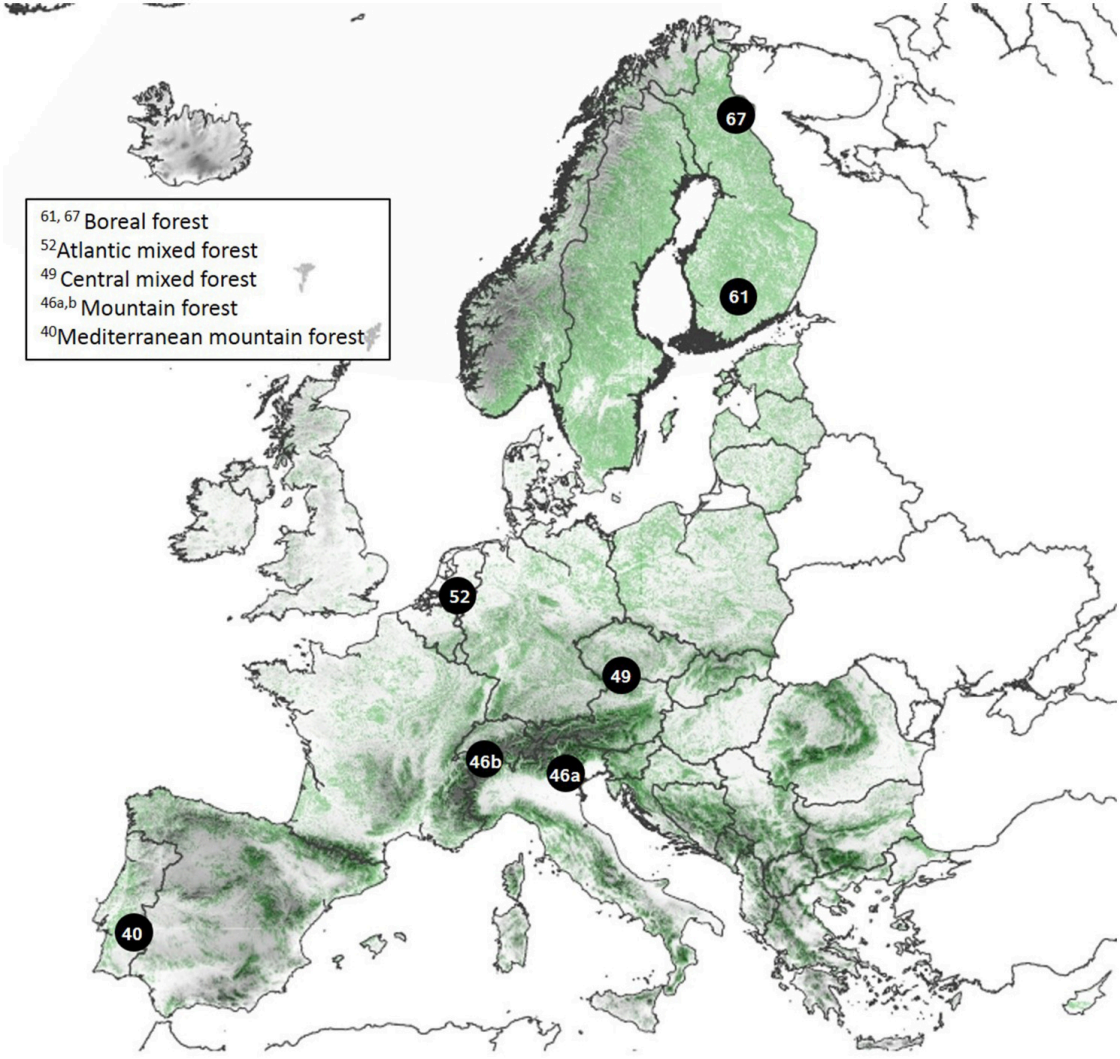
**Studied regions and their vegetation zones**. Each region is numbered by their northern latitude. The European map is based on CORINE Land Cover data with forests in green, and USGS digital elevation model.

We conducted a European wide study on Scots pine (*Pinus sylvestris* L.), Norway spruce (*Picea abies* (L.) Karst.), silver birch (*Betula pendula* Roth.) and common aspen (*Populus tremula* L.). These four species have a wide distribution and cover both deciduous angiosperm species and evergreen coniferous species. In total, we studied trees in seven regions along a climate gradient across Europe from northern Finland (67°N 29°E) to Portugal (40°N 7°W) (Figure 1, Table 1), and selected one moist soil site and one dry soil site per region based on soil type, ground vegetation, and soil moisture measurements. Measurements on needle lengths of *Pinus* and *Picea* showed that the needles from the moist soil sites were longer than the ones from the dry soil sites within each region with the exception of *Pinus* at the northern Finland (Figure S1 in the Supplementary Material). The climate gradient runs from cold and slow growth conditions in the north, through higher growth conditions at mid-latitudes, to drier and slower growth conditions in the south (Table 1).

**Table 1:** **Information on sampling regions, climate conditions (annual mean temperature; mean temperature for January; annual sum of precipitation; sum of precipitation for June, July and August; annual sum of potential evapotranspiration (PET) based on the Jensen-Haise method; sum of PET for June, July and August in years 1950–2000) and on sampled trees**.

**Region (latitude code)**	**Coordinates**	**Elevation, m.a.s.l**.	**Annual T, °C**	**Jan T, °C**	**Annual Precip.mm**	**JJA Precip.mm**	**Annual PET, mm**	**JJA PET, mm**	**Species**	**Site**	**No. of trees**	**Tree H, m**	**Mean sample age**
Finland North (67)	67°N 29°E	380	−2	−13	545	205	380	315	*Pinus sylvestris*	Moist	5	5–10	20
										Dry	5	7–11	18
	67°N 29°E	405	−2	−13	545	205	380	315	*Picea abies*	Moist	5	6–11	17
										Dry	5	6–11	16
	67°N 29°E	370	−2	−13	545	205	380	315	*Populus tremula*	Moist	5	7–11	11
										Dry	5	5–9	13
Finland South (61)	61°N 24°E	140	3	-−	610	210	585	405	*Pinus sylvestris*	Moist	5	10–13	10
										Dry	5	6–18	12
	61°N 24°E	140	3	−9	610	210	585	405	*Picea abies*	Moist	5	5–9	7
										Dry	4	9–11	9
	60°N 24°E	60	4	−7	645	200	645	425	*Betula pendula*	Moist	3	7–15	4
										Dry	3	6–7	3
	61°N 24°E	150	3	−9	610	210	585	405	*Populus tremula*	Moist	3	8–10	4
										Dry	5	6–9	6
Netherlands (52)	52°N 05°E	5	9	2	765	220	885	460	*Pinus sylvestris*	Moist	5	8–11	3
										Dry	5	9–17	4
	52°N 05°E	5	9	2	765	220	885	460	*Picea abies*	Moist	5	14–17	5
										Dry	5	16–21	7
	52°N 05°E	5	9	2	765	220	885	460	*Betula pendula*	Moist	4	17	8
										Dry	4	11–23	4
	52°N 05°E	5	9	2	765	220	885	460	*Populus tremula*	Moist	5	18–22	7
										Dry	5	8–24	6
Czech Republic (49)	49°N 16°E	416	8	−3	575	230	890	485	*Pinus sylvestris*	Moist	5	5–6	2
										Dry	5	7–9	4
	49°N 16°E	416	8	−3	575	230	890	485	*Picea abies*	Moist	5	6–9	2
										Dry	5	7–9	3
	49°N 16°E	416	8	−3	575	230	890	485	*Betula pendula*	Moist	3	7–8	1
										Dry	5	5–9	3
	49°N 16°E	416	8	−3	575	230	890	485	*Populus tremula*	Moist	5	8	4
										Dry	5	8–12	4
Italy (46a)	46°N 12°E	1075	7	−4	1070	350	765	430	*Pinus sylvestris*	Moist	5	6–10	4
										Dry	5	5–6	27
	46°N 12°E	1075	7	−4	1070	350	765	430	*Picea abies*	Moist	5	5–14	5
										Dry	5	7–10	8
Switzerland (46b)	46°N 08°E	645	9	0	660	170	910	480	*Pinus sylvestris*	Moist	5	18–21	12
										Dry	5	8–11	13
	46°N 08°E	1340	5	−3	1315	390	610	380	*Picea abies*	Moist	4	20–30	8
										Dry	5	14–21	13
Portugal (40)	40°N 07°W	1450	8	2	1740	125	820	450	*Pinus sylvestris*	Moist	–	–	–
										Dry	5	10–14	14
	40°N 07°W	1500	8	2	1725	125	885	455	*Betula pendula*	Moist	4	8–9	20
										Dry	5	8–14	14

Climate data is from WorldClim original 30-s data (http://www.worldclim.org/bioclim) downscaled to 100-m resolution (Zimmermann and Roberts, 2001) for all but the Finnish sites; the Italian sites and the Swiss pine site have their precipitation data from a nearby weather stations at San Vito di Cadore (Centre of Studies of Alpine Environment) and Sierre (http://www.meteoswiss.ch), respectively, due to highly varying topography. Mean sample age refers to the number of growth rings in the xylem tissue at the fixed 0.7-m-sampling distance from branch tip. Especially in the case of Betula pendula, some of the five sampled trees per species and per site were removed from the dataset due to inadequate sampling material for osmolality measurements.

For each region, we selected five trees per species and per moist and dry soil site (Table 1). We selected healthy trees more than 5 m in height to harvest one 0.7-m-long branch (linear distance from tip) that was fully exposed to light in order to avoid shading effects. Fixed distance from the branch tip was selected for sampling to fix the transport distance from the C source to the sampling location. Branches were cut at 1 pm or later in the afternoon to minimize the impact of confounding diurnal trends in osmolality. Two 5-cm-long branch segments between distances 70 and 60 cm from the branch tip were cut. The basipetal segment was put in 50% ethanol for anatomical analysis of phloem area. The acropetal segment was sealed in a plastic tube and frozen immediately in the field in liquid nitrogen or dry ice for osmolality and water content measurements. Sampling was performed in late summer of 2014 after the end of seasonal secondary growth but before leaf senescence in the different regions.

### Phloem osmolality

Phloem osmolality measurements were conducted in the laboratory at the University of Helsinki. Frozen samples were brought to room temperature for 15 min to thaw. Freezing and thawing the samples rapidly breaks the cell membranes and releases symplastic contents to the apoplast (Kikuta and Richter, 1992; Callister et al., 2006). The outer bark was scraped away with a razor blade and each sample cut in two 2 cm pieces in order to have two subsamples of each branch. The inner bark (including cambium and all the tissues from cambium to the innermost periderm) was separated from xylem on the basis of the hardness and color differences between the tissues and weighted for fresh mass (FM). The samples were set in silica-based membrane collection tubes (GeneJET Plasmid Miniprep Kit, Thermo Scientific, Massachusetts, USA) into a centrifuge (Heraeus Fresco 17, Thermo Scientific, Massachusetts, USA) at 14,000 g for 10 min (Devaux et al., 2009). The liquid was collected in osmometer tubes and the osmolality of the liquid was measured with a freezing-point osmometer (Osmomat-030 Freezing point osmometer, Gonotec, Berlin, DE).

We assumed that the ratio of phloem tissue volume to whole inner bark tissue volume is large enough that it is justified to refer to the collected inner bark sap as phloem sap.

### Amount of solutes and water content

In order to get comparable information of the accumulation of cellular solutes in secondary phloem in different tree individuals growing in different regions, phloem osmolality measurements either need to be analyzed at full tissue saturation (Rosner et al., 2001) or be connected with tissue water content measurements. To determine the amount of solutes (n) and water content (WC) in the inner bark tissue, we randomly selected three of the five phloem osmolality samples per species and site. The samples were dried at 80°C in an oven for 72 h to obtain their dry mass (DM). WC (g g^−1^_DM_) was calculated as the difference between FM and DM divided by the DM, and n (mol kg^−1^_DM_) was calculated as osmolality multiplied by WC.

### Non-structural carbohydrate composition

To avoid methodological artifacts (Quentin et al., 2015), all NSC measurements were done at the Natural Resources Institute Finland. Furthermore, to standardize our results, we focused on the ratio between starch and the total NSC content, and the ratio between the target sugar and the total soluble sugar content.

We analyzed NSC composition of evergreen conifer phloem by using a sub-sample (ca. 2 cm in length) of the segment collected for the measurements of osmolality and water content. NSC measurements were performed according to Jyske et al. (2015). Briefly, samples of inner bark were cut into matchstick-sized pieces, freeze-dried for 72 h, and milled with a ball-mill while kept frozen. About 20 mg of powder was weighed into glass test tubes and heated to 100°C to deactivate the enzymes. The soluble sugars were extracted twice (at 100°C) by using 80% ethanol to which m-erythtrit (Calbiochem, Merck KGaA, Darmstadt, Germany) was added as an internal standard. The sugar extracts were evaporated to dryness with nitrogen flow, silylated with 20% TMSI-pyridine mixture (i.e., 1-trimethylsilyl-imidazole; Sigma-Aldrich, Darmstadt, Germany), and analyzed with gas chromatography–mass spectrometry (GC-MS; Agilent Hewlett-Packard 6890 GC, equipped with a Zebron ZB-SemiVolatiles column (30 m × 0.25 mm i.d × 0.25 μm df) and Hewlett-Packard 5973 MSD, EI-MS 70 eV), in which helium was used as a carrier gas (flow 1.5 ml/min). The chromatographic conditions were as follows: initial temperature 110°C; rate of temperature increase 10°C min^−1^; final temperature 320°C maintained for 14 min; injector temperature 260°C, and split ratio 1:20. The MS-interface temperature was 300°C and ion source temperature was 230°C. In the analysis, the compounds were identified on the basis of their mass spectra and retention times as verified by using the following authentic compounds (i.e., external standards): D-fructose (Merck, Darmstadt, Germany), myo-inositol (Merck), D-glucose (BDH AnalaR, VWR International Ltd, Poole, UK), sorbitol (Fluka, Sigma-Aldrich), sucrose (BDH AnalaR), D-raffinose pentahydrate (Fluka). For pinitol, fructose was used as a standard. The results were calculated using an internal standard and the external standards.

The soluble-sugar-free samples obtained after extraction were used for starch analyses with a commercial starch assay kit (Total Starch Assay Procedure, Megazyme International, Wicklow, Ireland). Briefly, starch in residual pellets was hydrolyzed into maltodextrins by adding α-amylase (in MOPS-buffer, pH 7) and incubated for 6 min at 100.5°C. Next, the samples were suspended in acetate buffer (pH 4.5) and amyloglugosidase was added to hydrolyze maltodextrins into d-glucose by incubating for 30 min at 50.5°C. The absorbance of the samples was measured colorimetrically (Shimadzu UV-2401 spectrometer at 510 nm) using glucose oxidase and peroxidase. The standard curve was made with D-glucose (BDH AnalaR).

### Phloem area and sample age measurements

The most basipetal branch segment was used for anatomical analysis. Each segment infiltrated in 50% ethanol was cut with a hand saw to have a 5–8 mm thick disk. Disks were then dehydrated with immersions in ascending ethanol concentrations until absolute ethanol, infiltrated with liquid paraffin, and embedded into paraffin blocks (Anderson and Bancroft, 2002). The blocks were trimmed and moistened with cold water for at least 2 h to soften the woody tissue and then cut with a rotary microtome (RM2245, Leica). Sections (10–15 μm in thickness) were then stained with a solution of safranine and Astra blue (1 and 0.5% in distilled water, respectively), dehydrated with alcohol (50 and 96%), rinsed with xylol and permanently fixed by mounting a cover glass with Eukitt (Bioptica, Milan, Italy). Digital images were captured at 40 × magnifications with a camera mounted on a light microscope (Eclipse80i, Nikon) to cover the whole cross-sectional area and then stitched with PTGui v8.3.10 (New House Internet Services B.V., Rotterdam, The Netherlands). Stitched images were analyzed with ROXAS v2.1 (von Arx and Dietz, 2005; von Arx and Carrer, 2014) along a wedge of known angle centered at the pith to identify tree-ring boundaries and determine branch age. Proxy for the growth rate (cm year^−1^) of 70-cm-long branches could be calculated from the branch age. In addition, the non-collapsed phloem area was determined from the wedge and upscaled to the total cross-section (Zhang et al., 2015). Collapsed phloem was identified as the phloem older than 1 year, characterized by bigger and stretched cells.

### Statistical analysis

We first analyzed the effect of water content (WC) on osmolality. A two-level mixed-effect model for explaining osmolality was created. The fixed term of the model included the explanatory variables 1/WC, species and their interaction. In addition, the model had random intercepts for levels describing the nested structure of the data: regions, and sites within regions. Random intercepts followed normal distribution. These models, and mixed-effect models described below, were fitted with the function lme of the R package nlme (Pinheiro et al., 2013). All statistical analyses were performed with R version 3.2.2 (R Core Team, 2013).

Solute content—osmolality regression was fitted but the significance of the fit was not analyzed because the osmolality was used in the calculation of the solute content, thus creating dependency of response and explanatory variables. Nonetheless fitted curves are given to guide the reader's eye. Curves were estimated with the function nls of the R package stats (Bates and Chambers, 1992) assuming a relationship *y* = *a* + *x*^∧^*b* between ordinate (*y*) and abscissa (*x*). Parameters *a* and *b* were fitted by species.

Secondly, we compared the differences of osmolality, n, WC, and NSC composition between species and regions. Therefore, the fixed term of the mixed-effect model included species and region, and several covariates [sample age, non-collapsed phloem area, tree height, site moisture status (moist/dry)]. The random term included sites, and, in the case of osmolality, observations within tree as we had two repetitions per tree. First, we performed the selection of covariates using AIC criterion and step AIC-function of the R package MASS (Venables and Ripley, 2002) when the site moisture status, species, and region were always in the model. Second, also the site moisture status and species were removed from the model if they did not improve AIC. ANOVA results of the model in the Result section are shown for the marginal effects, i.e., for the effects, when all other variables are already in the model. Pairwise differences between regions and species were tested with Tukey's range test (R function glht—R package multcomp; Hothorn et al., 2008), except for NSC-related variables, where ANOVA results were used to test whether the conifers significantly differ from each other.

## Results

**Figure 2:**
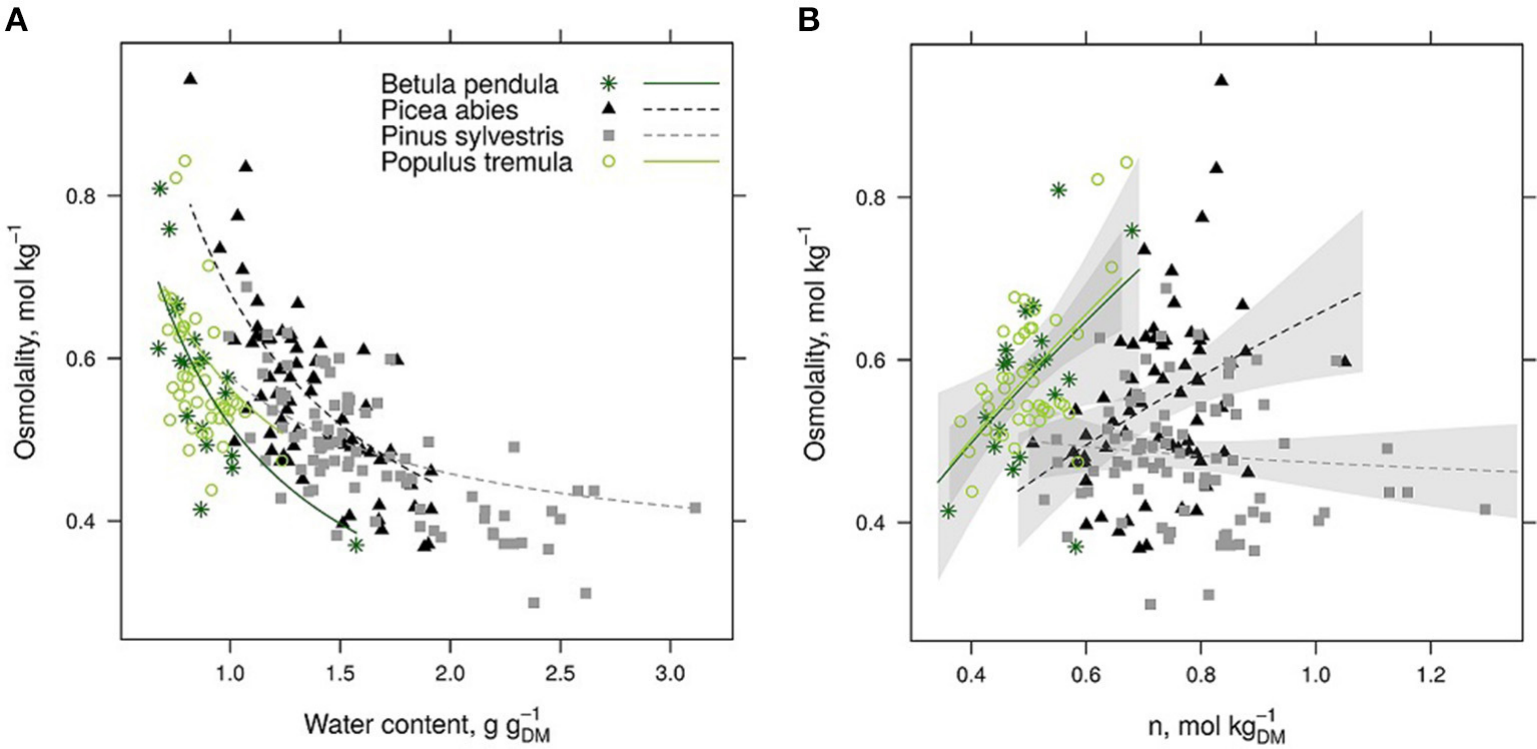
**Phloem osmolality is shown against tissue water content (A) and solute content (B) per tissue dry mass (***DM***) for each species**. Species-specific model fits are drawn in a based on a mixed-effect model (Table 2). In **(B)**, power fits and 95% confidence intervals are drawn for each species based on the raw data to guide the eye although statistical tests are not justified (*n* is not independent from osmolality).

Phloem osmolality decreased with increasing water content per tissue dry mass in all studied species (Table 2, Figure 2A). In addition to tissue water content, phloem osmolality increased with increasing tissue solute content (calculated from osmolality and water content measurements) in *Populus* and *Betula*, but such a trend was either weaker (*Picea*) or absent (*Pinus*) for both evergreen conifers (Figure 2B).

**Table 2:** **Mixed-effect model result for testing the effect of species, water content (WC) and their interaction on phloem osmolality**.

**Dependent variable**	**Independent variables**	**Class**	**Estimate ± SE**
Osmolality, mol kg^−1^	Intercept^***^	(*Betula pendula)*	0.15 ± 0.09
	Species^***^	*Pinus sylvestris*	0.19 ± 0.09
		*Picea abies*	0.04 ± 0.10
		*Populus tremula*	0.12 ± 0.12
	1 WC^−1^^***^	(*Betula pendula)*	0.36 ± 0.07^***^
	Species × 1 WC^−1^^*^	*Pinus sylvestris*	−0.13 ± 0.09
		*Picea abies*	0.12 ± 0.10
		*Populus tremula*	−0.07 ± 0.10

Betula pendula is used as reference for the model estimates for the class variable species. Sample size is 208.

* *P < 0.05*,

*** P < 0.001.

**Figure 3:**
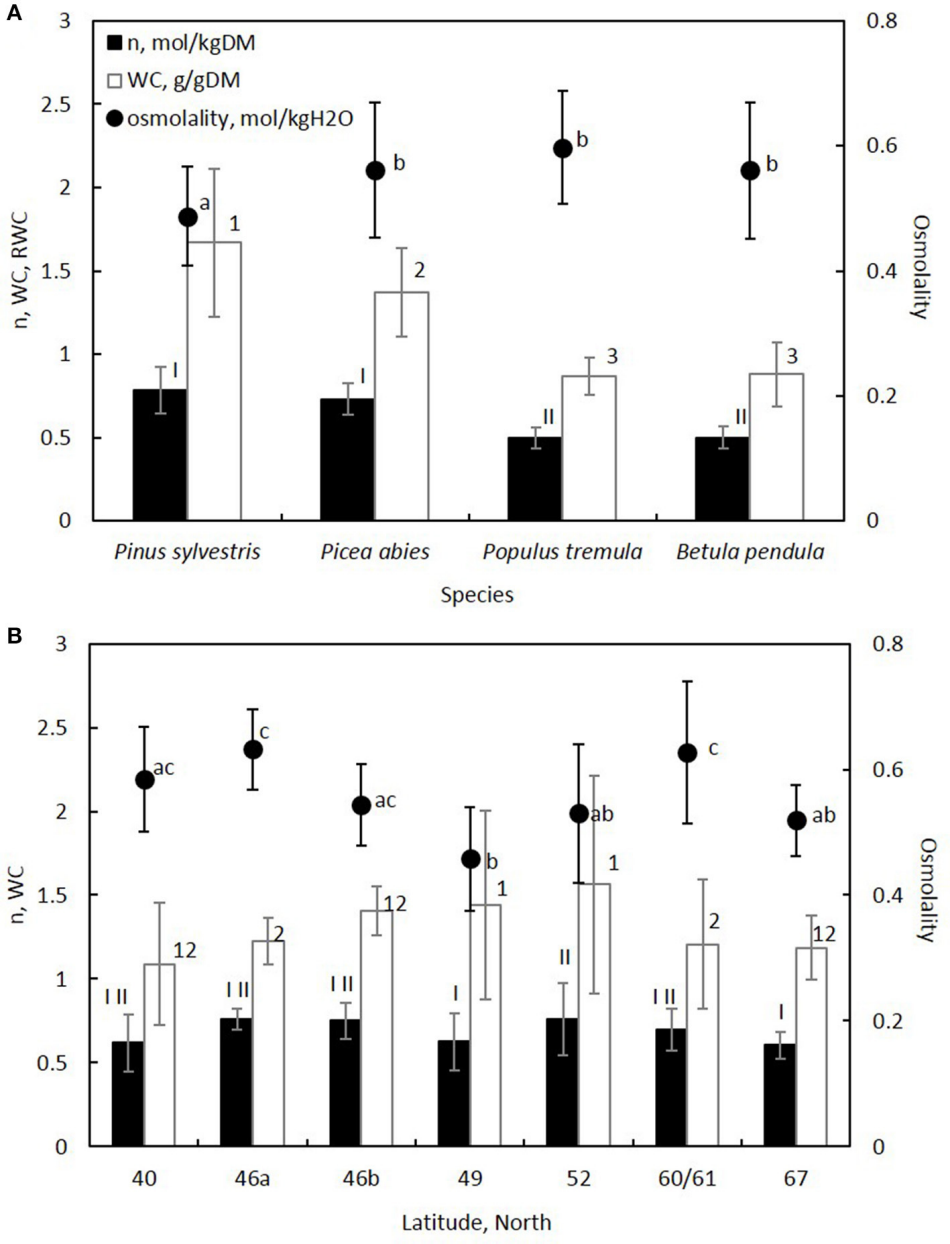
**Solute content (n) and water content (WC) per tissue dry mass, and osmolality of the tissue are shown for each (A) species and (B) region**. The latitudes in **(B)** represent countries as shown in Table 1 and Figure 1. Error bars indicate standard deviation. Significant differences between species and regions were analyzed with a mixed-effect model for n, WC and osmolality (Table 3), and are shown with different Roman numbers, Arabic numbers and letters, respectively.

Phloem osmolality (mol kg^−1^) varied across regions between 0.38–0.60 (*Pinus*), 0.44–0.69 (*Picea*), 0.53–0.69 (*Populus*), and 0.49–0.64 (*Betula*). Phloem osmolality was on average 15% lower in *Pinus* than in the other species (Table 3, Figure 3A). Among the studied regions, osmolality was lowest at mid-latitude (Czech Republic), increased toward the north and the south with the highest average values being measured in Southern Finland and Italy, respectively, and then decreased again in Northern Finland and Portugal (Table 3, Figure 3B). The difference in the average osmolality between the Czech Republic and Southern Finland was 37%, and between the Czech Republic and Italy 38%. There was no significant difference in phloem osmolality between dry and moist soil sites within the regions. Tree height, sample age or, the area of non-collapsed phloem were not related to phloem osmolality. The latitudinal trends were visible in all species (Figure S2 in the Supplementary Material).

**Table 3:** **Mixed-effect model results for testing the influence of species and region on osmolality, water content (WC), and solute content (n)**.

**Dependent variable**	**Covariates and fixed effects**	**Class**	**Estimate ± SE**
Osmolality, mol kg^−1^	Intercept^***^	(dry site, *Betula pendula*, 40°N)	0.64±0.03^***^
*N* = 342	Site	Moist site	−0.02±0.02
	Species^***^	*Pinus sylvestris*	−0.081±0.028^**^
		*Picea abies*	0.002±0.029
		*Populus tremula*	0.067±0.031^*^
	Region^***^	46a°N	0.03±0.05
		46b°N	−0.04±0.05
		49°N	−0.17±0.04^***^
		52°N	−0.09±0.04^*^
		61°N	0.02±0.04
		67°N	−0.10±0.04^*^
WC, g g^−1^_DM_	Intercept^***^	(dry site, *Betula pendula*, 40°N)	0.90±0.14^***^
*N* = 208	Sample age, y^*^		−0.0074±0.0030^*^
	Non-c. phloem area, mm^2^^**^		0.016±0.005^**^
	Tree height, m^*^		−0.01±0.005^*^
	Site	Moist site	0.13±0.07
	Species^***^	*Pinus sylvestris*	0.85±0.11^***^
		*Picea abies*	0.55±0.11^***^
		*Populus tremula*	−0.01±0.12
	Region^**^	46a°N	−0.33±0.18
		46b°N	−0.03±0.18
		49°N	0.12±0.16
		52°N	0.23±0.16
		61°N	−0.20±0.16
		67°N	−0.11±0.17
n, mol kg^−1^_DM_	Intercept^***^	(*Betula pendula*, 40°N)	0.57±0.05^***^
*N* = 208	Sample age, y^***^		−0.005±0.001^***^
	Species^***^	*Pinus sylvestris*	0.31±0.04^***^
		*Picea abies*	0.25±0.04^***^
		*Populus tremula*	0.01±0.04
	Region^*^	46a°N	−0.030±0.06
		46b°N	−0.030±0.06
		49°N	−0.092±0.05
		52°N	0.023±0.05
		61°N	−0.033±0.05
		67°N	−0.090±0.05

Potential covariates in the model were site moisture status, tree height, sample age and non-collapsed phloem area; covariates and their order in the final model were selected with AIC. Dry site, Betula pendula and Portugal (40°N) are used as references for the model estimates for the class variables site, species and region, respectively, in the model output. N is sample size.

* *P < 0.05*,

** *P < 0.01*,

*** P < 0.001.

Water content per dry mass was on average 91% higher in *Pinus* and 57% higher in *Picea* in comparison to the deciduous angiosperm species (Figure 3A). The highest tissue water content was measured at mid-latitudes, from where it decreased on average by 21% toward northern latitudes and 28% toward southern latitudes (Figure 3B). Tissue water content variability was high at the intermediate latitudes (Figure 3B). In addition to species and region, increasing non-collapsed phloem area increased water content per tissue dry mass indicating that non-collapsed phloem contains more water in comparison to collapsed phloem (Table 3). Water content per tissue dry mass was higher in younger samples in comparison to older samples, and higher in shorter trees in comparison to taller trees (Table 3). Phloem water content was slightly higher in moist soil sites in comparison to dry soil sites, but this difference was not statistically significant (Table 3).

Solute content per phloem dry mass was on average 51% higher in the studied evergreen conifers in comparison to the deciduous angiosperm species (Figure 3A). Solute content showed only few statistically significant differences between the regions (Table 3, Figure 3B), and a decreasing trend with increasing sample age (Table 3).

**Figure 4:**
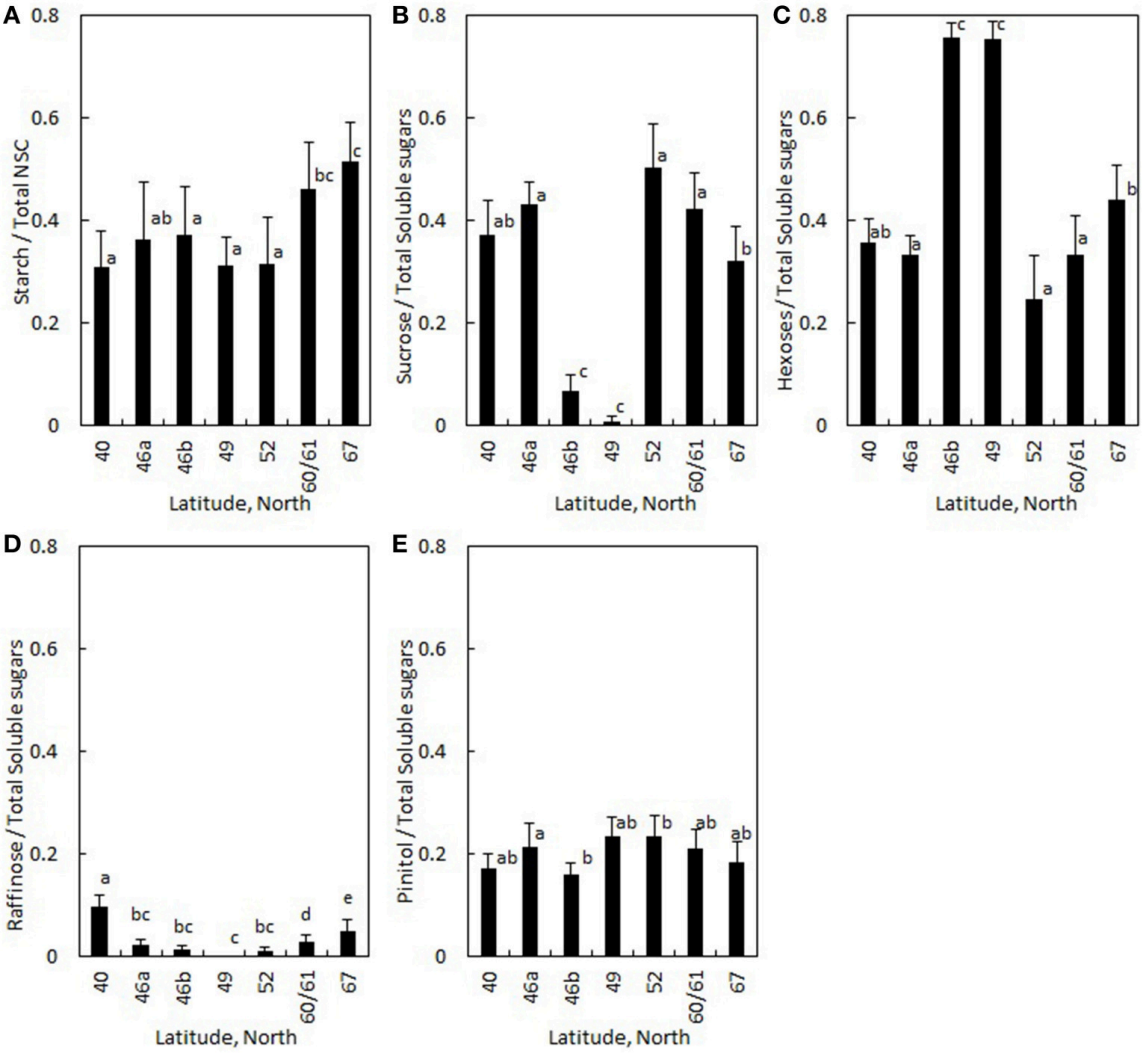
**Ratio of (A) starch to NSC and ratio of (B) sucrose, (C) hexoses (i.e., glucose + fructose), (D) raffinose and (E) pinitol to total soluble sugars averaged for ***Pinus sylvestris*** and ***Picea abies*** is plotted in different regions**. The latitudes represent countries as shown in Figure 1 and Table 1. Error bars indicate standard deviation. Significant differences between regions were analyzed with a mixed-effect model (Table 4), and are shown with different letters.

The ratio of starch to total NSC increased approximately 60% from Portugal to the Finnish regions (Table 4, Figure 4A). The ratio of disaccharide sucrose to total soluble sugars was the lowest at mid-latitudes in Switzerland and the Czech Republic (Table 4, Figure 4B), whereas the ratio of monosaccharides glucose and fructose (i.e., hexoses) to total soluble sugars was the highest in these regions (Table 4, Figure 4C). Raffinose content was negligible at mid-latitudes in Czech Republic, and increased with both increasing and decreasing latitudes (Table 4, Figure 4D). The ratio of raffinose to all soluble sugars was the only soluble sugar that had significantly different values in the two studied evergreen conifers: the share of raffinose was 10% higher in *Pinus* in comparison to *Picea* (Table 4). In contrast, the share of pinitol to total soluble sugars showed only a few statistically significant differences across regions and there was no difference between the two conifers (Table 4, Figure 4E). No significant differences were observed in any soluble sugars between moist and dry soil sites (Table 4). Similar latitudinal trends were visible in NSC composition in absolute concentrations (Figure S3 in the Supplementary Material).

**Table 4:** **Mixed-effect model results for testing the influence of species and region on the ratio of starch to non-structural carbohydrates (NSC), ratio of sucrose, hexoses (i.e., glucose + fructose), raffinose and pinitol to total soluble sugars**.

**Dependent variable**	**Covariates and fixed effects**	**Class**	**Estimate ± SE**
Starch/NSC	Intercept^**^	(40°N)	0.18±0.06 ^**^
*N* = 86	Sample age, y^*^		0.001±0.001
	Tree height, m^**^		0.0071±0.0023^**^
	Region^***^	46a°N	0.11±0.06
		46b°N	0.05±0.06
		49°N	0.08±0.06
		52°N	0.02±0.06
		61°N	0.20±0.06 ^**^
		67°N	0.26±0.06 ^**^
Sucrose/solubles	Intercept^***^	(40°N)	0.23±0.05 ^***^
*N* = 86	Sample age, y^*^		0.0026±0.0010^*^
	Non-c. phloem area, mm^2^^**^		0.0055±0.0016^**^
	Tree height, m^**^		0.0045±0.0017^**^
	Region^***^	46a°N	0.10±0.05
		46b°N	−0.30±0.05 ^***^
		49°N	−0.28±0.05 ^***^
		52°N	0.14±0.05^*^
		61°N	0.10±0.05
		67°N	−0.02±0.05
Hexoses/solubles	Intercept^***^	(40°N)	0.43±0.05 ^***^
*N* = 86	Non-c. phloem area, mm^2^^*^		−0.0036±0.0017^*^
	Tree height, m^**^		−0.0046±0.0017^**^
	Region^***^	46a°N	−0.04±0.05
		46b°N	0.43±0.05 ^***^
		49°N	0.38±0.05 ^***^
		52°N	−0.07±0.05
		61°N	−0.04±0.05
		67°N	0.08±0.05
Raffinose/solubles	Intercept^***^	(dry site, *Picea abies*, 40°N)	0.08±0.01 ^***^
*N* = 86	Tree height, m^*^		0.0010±0.0004^*^
	Site	moist site	−0.006±0.004
	Species^*^	*Pinus sylvestris*	0.0081±0.0037^*^
	Region^***^	46a°N	−0.07±0.01 ^***^
		46b°N	−0.09±0.01 ^***^
		49°N	−0.10±0.01 ^***^
		52°N	−0.09±0.01 ^***^
		61°N	−0.07±0.01 ^***^
		67°N	−0.04±0.01 ^***^
Pinitol/solubles	Intercept^***^	(dry site, 40°N)	0.21±0.03 ^***^
*N* = 86	Sample age, y^***^		−0.0023±0.0006^^***^^
	Non-c. phloem area, mm^2^		−0.0017±0.0010
	Site	moist site	0.019±0.010
	Region^*^	46a°N	0.032±0.027
		46b°N	−0.022±0.027
		49°N	0.026±0.027
		52°N	0.038±0.027
		61°N	0.028±0.027
		67°N	−0.002±0.027

Potential covariates in the model were site moisture status, tree height, sample age and non-collapsed phloem area; covariates and their order in the final model were selected with AIC. Dry site, Picea abies and Portugal (40°N) are used as references for the model estimates for the class variables site, species and region, respectively, in the model output. N is sample size.

* *P < 0.05*,

** *P < 0.01*,

*** P < 0.001.

In addition to region, the ratios of starch to total NSC, and the ratios of sucrose and raffinose to all soluble sugars were positively linked to tree height (Table 4). The ratio of starch to total NSC increased with increasing sample age (Table 4). Similarly, sample age affected the share of sucrose positively, as did the area of non-collapsed phloem (Table 4). Share of hexoses, on contrary, decreased with increasing tree height, and was the lower the higher the area of non-collapsed phloem (Table 4). The share of pinitol decreased with increasing sample age (Table 4).

Branch growth rate was highest at mid-latitudes and decreased toward north and south (Table 1). Also needle length in the studied conifers showed similar trend (Figure S1 in the Supplementary Material).

## Discussion

### Latitudinal trends and species differences in osmolality

The results showed that the major determinant of observed variation in phloem osmolality across Europe was tissue water content instead of solute content, in contrary to what we expected. Solute content played a role in explaining the variation in phloem osmolality for both deciduous angiosperm species (*Betula pendula* and *Populus tremula*), but its effect was weak for the two evergreen conifers (*Pinus sylvestris* and *Picea abies*). We thus found support for active osmoregulation, by adjusting sugar contents, for deciduous angiosperms, but not for evergreen conifers. Phloem transport, conversion of NSC from one form to another, or unloading of sugars with the xylem may contribute to such osmoregulation.

The study confirmed our hypothesis that phloem osmolality increases from mid-latitudes toward the extreme ends of the latitudinal gradient following decreasing branch growth rate (see Table 1). A higher osmolality of phloem sap was expected in the driest conditions as it contributes to maintaining turgor when tree water potential is low, and in cold conditions because it decreases the freezing point of living tissue (Charrier et al., 2013a) and maintains sufficient metabolism as the metabolic efficiency decreases at lower temperatures (see e.g., Piper et al., 2006). Moreover, high osmolality may enable refilling of xylem conducting elements embolised during freezing and thawing, as has been shown for *Juglans regia* (Charrier et al., 2013b) and *Picea abies* in alpine timberline (Mayr et al., 2014).

Local soil properties and/or topography had no effect on phloem osmolality, implying that climate rather than soil water supply affected phloem osmolality and its components. The sites where selected subjectively, but the contrast between two moisture statuses was strong enough to induce differences in needle length of *Pinus* and *Picea* (see Figure S1 in the Supplementary Material). Although site moisture status did not have a direct effect on phloem osmolality or its components, it played a role via sample age and non-collapsed phloem area as these variables varied between moist and dry soil sites. These sources of variation were controlled in the analyses with statistical tests.

The latitudinal trends observed in phloem osmolality followed the latitudinal trends observed in the level of tissue water content, whereas the level of phloem solute content was surprisingly similar across Europe. Low phloem water content per tissue dry mass in the extreme ends of the latitudinal gradient can either be caused by differences in weather conditions during sampling, phloem tissue anatomy, or elastic tissue adjustment. However, the weather cannot explain the whole water content variability, e.g., the low phloem water content observed in the two most northern regions were measured on rainy days. Phloem anatomy is one potential explanation although the literature is scarce concerning the differences in phloem anatomy between different climates (see Gričar et al., 2015); in xylem it is known that density typically increases with decreasing growth rate (e.g., Raiskila et al., 2006), and growth rate was indeed lower in the most extreme ends of the latitudinal gradient in comparison to the middle latitudes (see Table 1). Another potential explanation is latitudinal differences in tissue elasticity. It is known that elastic shrinkage and extraction of water is characteristic for phloem tissue both during cold (Zweifel and Häsler, 2000; Améglio et al., 2001; Lintunen et al., 2015) and drought (Zweifel et al., 2001; Steppe et al., 2006; Mencuccini et al., 2013) stress. Similarly, Gross and Koch (1991) and Callister et al. (2008) studied seasonal changes of leaf osmotic potential at full turgor in *Picea abies* and three *Eucalyptus* species, respectively, and concluded that the observed increase in leaf osmotic concentration in winter was mainly caused by decreased tissue water content (due to increased tissue elasticity) instead of active accumulation of solutes. On the other hand, previous studies on leaf osmotic potential under drought stress suggests that the turgor loss point in leaves is dictated by solute content via osmoregulation rather than elastic adjustment with tissue water content (Bartlett et al., 2014; Delzon, 2015; Maréchaux et al., 2015).

### Latitudinal trends and species differences in NSC composition

Although phloem solute content was rather stable between regions along the latitudinal gradient, the composition of the non-structural carbohydrate concentration (NSC) differed between regions. The proportion of starch to total NSC was 46% higher in the two regions measured in Finland in comparison to other regions. These results are in accordance with the study of Hoch and Körner (2012) on late-season NSC concentration in various tree species in the tree line ecotones, where they reported increasing NSC concentration in branch wood with elevation due to increased starch content. The results of our study and the study of Hoch and Körner (2012) imply that the high proportion of starch reflects the build-up of osmotically inactive C reservoir to balance between periods of low C supply, and to buffer during the events of abiotic stress (Sala et al., 2012; Hartmann, 2015). Starch is typically converted into soluble sugars as a result of decreasing temperature (Améglio et al., 2004), so high starch ratio measured in the north at the end of the growing season may lead to increased soluble sugar concentrations and phloem osmolality for winter. This conclusion is in accordance with earlier studies showing that starch concentration in xylem of *Picea abies* (Hou, 1985) and cambium of *Picea mariana* (Deslauriers et al., 2014) increase in autumn, whereas Jyske et al. (2015) found contradictory results for the inner bark of *Picea abies* where the ratio of starch to total NSC decreased from the mid-summer toward autumn. Especially *Pinus* species are known to store also lipids in their woody parts in high elevations/latitudes (Hoch et al., 2002; Hoch and Körner, 2003) which also contribute to C storage dynamics in case when C fixation is extremely constrained (Hoch and Körner, 2003).

Sucrose comprised only a minor share of total soluble sugars in the middle latitudes of Switzerland and the Czech Republic, whereas hexoses represented higher proportion in comparison to other regions. Sucrose is a non-reducing sugar, it is chemically stable, and has higher molar mass in comparison to other sugars such as hexoses glucose and fructose. The high hexose to sucrose ratio in the middle latitudes might be linked to mobilization of starch after an active growing period (Witt and Sauter, 1994; Deslauriers et al., 2014) or to high respiratory losses (Strimbeck et al., 2008). Similarly, Deslauriers et al. (2014) measured sucrose levels close to zero from *Picea mariana* xylem and cambium during July in Quebec, and suggested that growth activities might be the cause. On the other hand, high sucrose levels have been empirically connected to high cold tolerance in *Pinus sylvestris* and *Picea abies* needles (Aronsson et al., 1976; Strimbeck et al., 2008) and in *Picea abies* buds (Lipavská et al., 2000). Sucrose is known to be able to retain the liquid-crystalline state of membranes under osmotic stress caused by cold, drought, and salinity (see Lipavská et al., 2000). Also, it cannot be totally excluded that the seasonal timing of sampling was not fully synchronized between regions (i.e., trees might have been in different phenological phases between regions regarding e.g., the end of secondary growth, leaf senescence, cold acclimation, etc.). In general, sucrose is considered as the most important compound being translocated in phloem elements (Pate, 1976) and it is a preferable phloem transport sugar (Lang, 1978; Aoki et al., 2012; De Schepper et al., 2013), whereas hexoses are used more for storage and are present in high amounts in all living cells.

Our hypothesis that the stress-related soluble sugars raffinose and pinitol have high share of total soluble sugar content in the most drought and cold stressed environments was confirmed for raffinose, but not for pinitol. The proportion of raffinose from all soluble sugars was higher in the most southern and northern latitudes compared to the middle latitudes as we hypothesized. Accordingly, the role of raffinose in plant cell protection during environmental stress, such as drought and low temperatures is supported by several studies (e.g., Zuther et al., 2004; Nishizawa-Yokoi et al., 2008; dos Santos et al., 2011; Deslauriers et al., 2014), but is not yet demonstrated for the secondary phloem at continental scale. Deslauriers et al. (2014) reported raffinose concentration to increase in response to drought in the cambium and xylem of *Picea mariana* and concluded the osmoregulatory response to be directly dependent on raffinose. Jyske et al. (2015) reported increases in raffinose content of *Picea abies* phloem when approaching dormant season in the south and north of Finland. Accordingly, Simard et al. (2013) found raffinose concentration to increase during winter acclimation in the cambium of *Picea abies* and *Larix decidua* and Hoch et al. (2002) in the wood of *Pinus cembra*. The important role of raffinose among the soluble sugars in stressful environments is that it acts as osmoprotectant and antioxidant: raffinose protects cellular structures and sustains osmotic balance in plants (dos Santos et al., 2011), inhibits the oxidation of other molecules thus protecting plant cells from oxidative damage and maintaining redox homeostasis (Nishizawa-Yokoi et al., 2008), and inhibits the tendency of sucrose to crystallize and hence to lose its protective effect under stress conditions (Caffrey et al., 1988; Lipavská et al., 2000).

Surprisingly, the proportion of pinitol from all soluble sugars was not higher in the most northern and southern regions in comparison to other regions as hypothesized. This was against our hypothesis and is contradicting previous studies on several herbaceous species (e.g., Guoa and Oosterhuis, 1997) and tree foliage (e.g., Griffin et al., 2004), which described pinitol as sugar that increase tolerance to stress by drought, salinity or low temperature (Orthen et al., 1994). However, pinitol content in wood is higher during active growth than during cold (Hoch et al., 2002; Simard et al., 2013) or drought periods (Deslauriers et al., 2014), which implies that pinitol dynamics in the woody parts of trees differ from such dynamics in tree foliage or herbaceous species. In general, raffinose and pinitol were only present in small absolute amounts in the phloem, in agreement with other studies (e.g., Simard et al., 2013).

The ratio of raffinose to total soluble sugars was higher in *Pinus* than *Picea*. Any other soluble sugar did not show differences between the studied conifers. Raffinose concentrations have been measured previously in the inner bark of *Pinus sylvestris* (e.g., Antonova and Stasova, 2006) and *Picea abies* (Jyske et al., 2015), but there does not seem to be earlier studies allowing to compare the concentrations between the two species growing in the same region (see Quentin et al., 2015). *Picea abies* and *Pinus sylvestris* are both cold-tolerant tree species, but *Pinus* is better adapted to dry growth conditions. In general, both tissue water content and solute content expressed per tissue dry mass are higher in the studied evergreen conifers than in the deciduous angiosperm species, which might indicate higher phloem tissue density in the angiosperm species.

### Effect of branch age, tree height and non-conducting phloem area

Our sampling was done at a fixed distance of 70 cm from the branch tip, and thus the data represents a large variation in the age of the sample and area of non-collapsed phloem. There was a clear gradient in sample age from approximately 15 years in the cold Northern Finland and dry Portugal to approximately 3 years in the Czech Republic with good growing conditions. In the phloem of older samples, there was less water and solutes per phloem tissue dry mass. Thus, the lack of age-dependency of phloem osmolality could likely be explained by these two effects canceling each other out. Rosner et al. (2001) similarly found that differences in phloem water content parameters observed in *Picea abies* were explained by age-dependent structural changes in secondary phloem.

Also the ratios of sucrose and pinitol in the secondary phloem were dependent on branch age: the ratio of sucrose to total soluble sugars increased with branch age, whereas the ratio of pinitol decreased. Similarly Ericsson (1979) has shown that the youngest needles in *Pinus sylvestris* have more pinitol in comparison to older needles. The high levels found in the developing current-year needles as well as in the young phloem indicate that pinitol may also be involved in the synthesis of new cell components (Ericsson, 1979).

Tissue water content decreased with increasing tree height. However, the nature of this relationship is not easy to interpret as while tree height varied across regions and sites, height of branch cut was more homogeneous. Xylem conduits widen from stem/branch apex basally at nearly fixed rate (Anfodillo et al., 2013), therefore samples collected at fixed distance from the branch tip were virtually characterized by similar path-length hydraulic resistance (Petit and Anfodillo, 2009) and similar drop in water potential, if leaf transpiration is assumed to be comparable.

Increasing non-collapsed phloem area increased phloem water content indicating that conductive phloem contains more water in comparison to collapsed phloem. This result is in line with the study of Rosner et al. (2001) showing that phloem water content in *Picea abies* was clearly higher in non-collapsed, conducting phloem than in collapsed, non-conducting phloem. Similarly in *Quercus robur*, magnetic resonance imaging showed that phloem water content was the highest in the conductive phloem cells (De Schepper et al., 2012). This can be attributed to the degeneration of sieve cells and Strasburger cells (Rosner et al., 2001; De Schepper et al., 2012). Also the share of sucrose and raffinose to total soluble sugars increased with increasing non-collapsed phloem, whereas the share of hexoses decreased.

## Conclusion

Our study was the first one presenting patterns in osmolality and NSC composition in branch phloem at continental scale. It shows that osmolality increases with stronger drought or cold stress, and that both evergreen conifers and deciduous angiosperms adjust their phloem osmolality mainly by using water while solute content is surprisingly constant across large climate gradient. This suggests that passive, elastic adjustment with phloem water content occurs in stem and branches rather than osmoregulation. This is reasonable, as phloem is close to water potential equilibrium with the xylem (Thompson and Holbrook, 2003), which is achieved by exchanging water between these two tissues. Moreover, the composition of soluble sugars indicate that controlling raffinose, but not pinitol, allows trees to resist colder and drier conditions. The starch levels show that boreal cold stressed trees store more starch to survive the long cold winter. Overall, climate together with branch age and non-conducting phloem area rather than local soil water supply affected phloem osmolality and its components.

## Author contributions

Data from different countries was collected in a framework of topic groups 1 and 6 (COST STReESS, FP1106). FS coordinated the experimental design of plots across Europe. TP and SR made the osmolality measurements in the laboratory, TJ made the NSC measurements, GP had the main responsibility of the anatomy measurements, and GV and RLP made the needle length analysis presented in the supplementary material. MP and AL were responsible for the statistical analysis. AL and TH formulated the study questions with the help of the other authors. AL wrote most of the manuscript with the aid of TH, but all authors contributed by discussing and reviewing drafts and the final version.

## Funding

This article is based upon work from COST Action FP1106 STReESS, supported by COST (European Cooperation in Science and Technology). Financial support from the Academy of Finland was received by AL, TP, and TH (268342, 272041), and YS (1284701). TJ was supported by the Japan Society for the Promotion of Science (JSPS KAKENHI no. 26•04395) and MP by EU Life Programme (LIFE12 ENV/FI/000409). GV was supported by a grant from the Swiss State Secretariat for Education, Research and Innovation SERI (SBFI C14.0104). RG and JU were supported by Internal Grand Agency of Mendel University (IGA MENDELU 73/2013) and by the Ministry of Education, Sports and Youth of the Czech Republic (COST LD13017). EMRR is funded by the European Union's Horizon 2020 research and innovation programme under the Marie Sklodowska-Curie grant agreement No 659191 and by the Research Foundation—Flanders (FWO, Flanders, Belgium).

### Conflict of interest statement

The authors declare that the research was conducted in the absence of any commercial or financial relationships that could be construed as a potential conflict of interest.
