# Supplementary material for: Osmolality and Non-Structural Carbohydrate Composition in the Secondary Phloem of Trees across a Latitudinal Gradient in Europe
Source: Front Plant Sci. 2016 Jun 1;7:726. doi: 10.3389/fpls.2016.00726 (PMC4887491; doi:10.3389/fpls.2016.00726)
Supplement: Supplementary file 1 [file Presentation1.PDF]

SUPPLEMENTARY MATERIAL

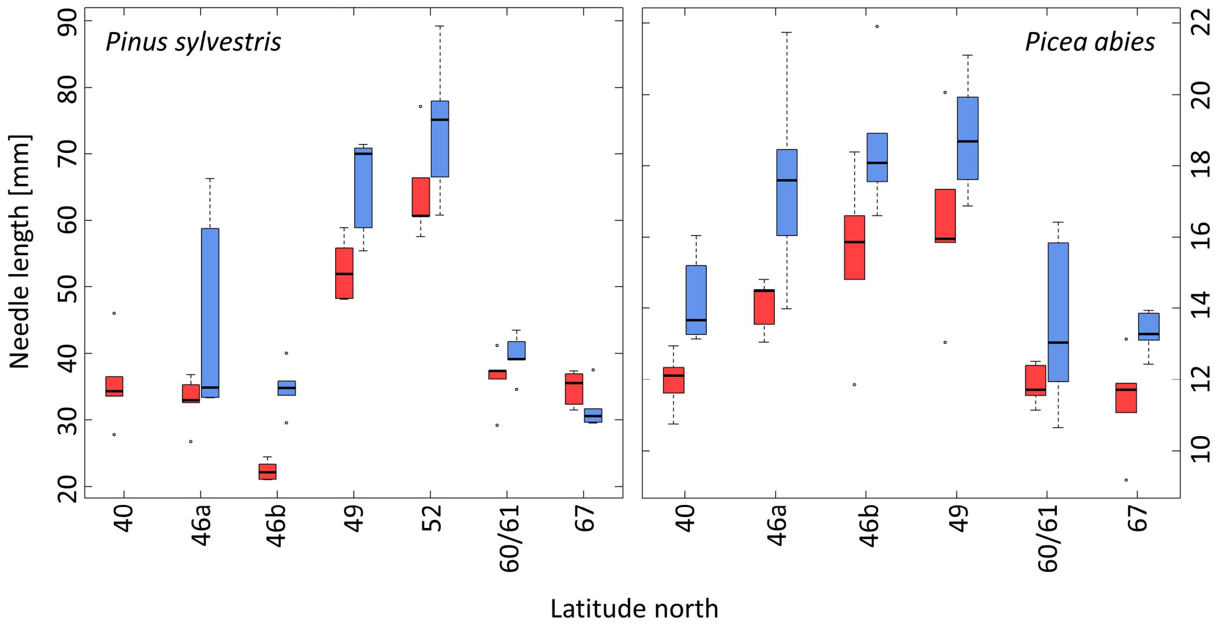

Figure S1. Needle length of *Pinus sylvestris* and *Picea abies* across the European gradient from south to north and how it differs between the local dry (red) and moist (blue) sites. Each box includes the average needle length from five branches (*n*=5). In both species, needles were significantly (*P*<0.05, Wilcoxon-test) shorter at the dry compared to the moist sites (*P. sylvestris*: -18.8%, *P. abies*: -15.5%). Only in north Finland (67), this relationship was inversed in *P sylvestris*.

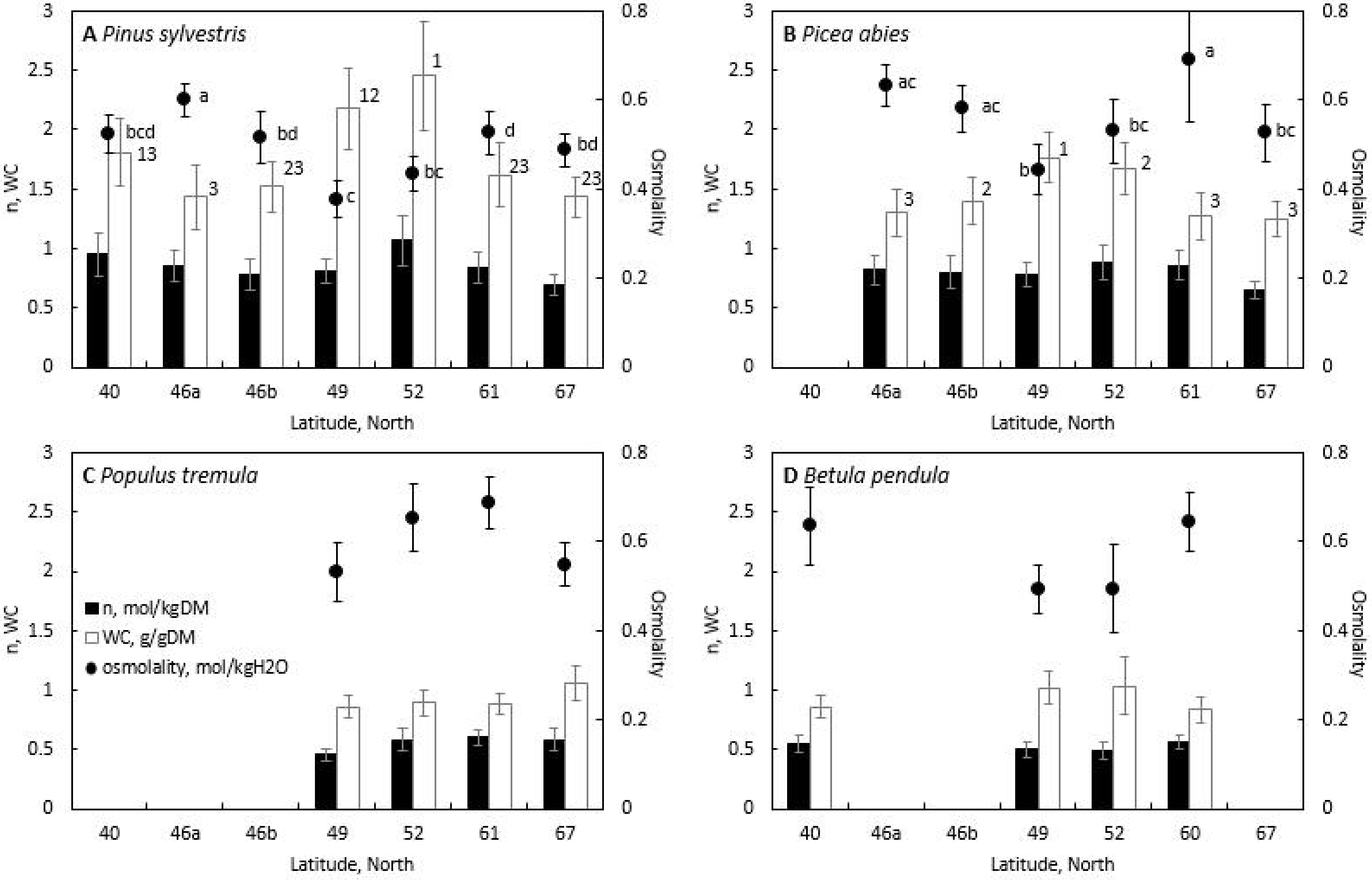

Figure S2. Solute content (n) and water content (WC) per tissue dry mass, and osmolality of the tissue are shown for each species (A-D) and region. The latitudes represent countries as shown in Table 1 and Figure 1. Error bars indicate standard deviation. Significant differences between species and regions were analyzed with a mixed-effect model for n, WC and osmolality, and are shown with different Roman numbers, Arabic numbers and letters, respectively; If they are not shown, latitude did not explain significantly the studied variable in the species-specific model.

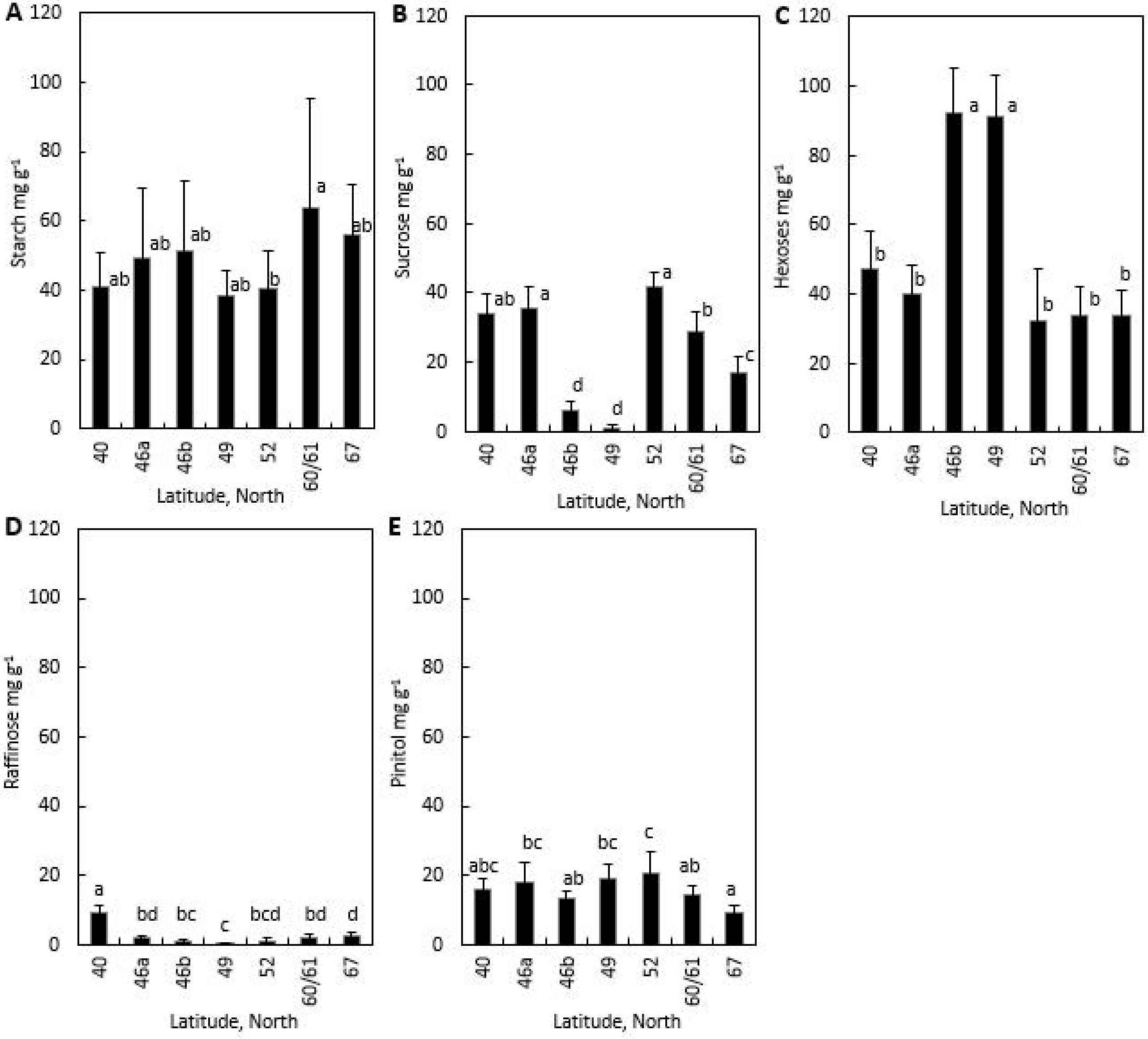

Figure S3. (A) Starch, (B) sucrose, (C) hexoses (i.e. glucose + fructose), (D) raffinose and (E) pinitol content per dry mass averaged for Pinus sylvestris and Picea abies is plotted in different regions. The latitudes represent countries as shown in Figure 1 and Table 1. Error bars indicate standard deviation. Significant differences between regions were analyzed with a mixed-effect model, and are shown with different letters.
